# High prevalence of pulmonary abnormalities in adults with oesophageal atresia: findings from a structured follow-up programme

**DOI:** 10.1136/bmjresp-2026-004434

**Published:** 2026-11-09

**Authors:** Stephanie A E Wintels, Julian F A van de Ven, Pierluigi Ciet, Lidewij S Visser, Arnold J F Duinisveld, Hanneke IJsselstijn, Rene M H Wijnen, John Vlot, Lieke S Kamphuis

**Affiliations:** 1 Department of Paediatric Surgery, Erasmus MC - Sophia Children’s Hospital, Rotterdam, The Netherlands; 2 Department of Pulmonology, Erasmus Medical Center, Rotterdam, The Netherlands; 3 Department of Radiology and Nuclear Medicine, Erasmus MC - Sophia Children’s Hospital, Rotterdam, The Netherlands; 4 Department of Radiology, University of Cagliari, Cagliari, Italy

**Keywords:** Bronchiectasis, Imaging/CT MRI etc, Respiratory Function Test

## Abstract

**Introduction:**

Respiratory comorbidities are common in adults with oesophageal atresia (OA), yet this group remains under-recognised in respiratory medicine. Despite guidelines advocating for structured pulmonary follow-up—including pulmonary function tests (PFTs) and chest CT imaging—implementation remains inconsistent. We aimed to assess respiratory outcomes in relation to anatomical abnormalities in a cohort of adult patients with OA to evaluate the need for standardised pulmonary surveillance.

**Methods:**

This retrospective cross-sectional study included adults with OA enrolled in the Erasmus Medical Center transition and follow-up programme between 2019 and 2022. All patients underwent pulmonology consultation, PFTs and chest CT imaging.

**Results:**

Of 182 patients (mean age 33±11 years, 9% OA type A, 85% OA type C), 128 patients (71%) showed abnormalities on CT imaging. Findings included bronchiectasis (12%), tracheomalacia (39%) and tracheal diverticulum (79%). 83 patients (46%) reported respiratory symptoms, most frequently dyspnoea (31%), cough (27%) and sputum production (11%). Symptom burden was associated with CT abnormalities (p=0.022). Interestingly, 62% of asymptomatic patients showed structural changes on chest CT. Bronchiectasis was more prevalent in patients reporting cough and/or sputum than in asymptomatic individuals (21% vs 9%, p=0.019). Mean prebronchodilator PFT z-scores (±SD) were: total lung capacity −1.34 (±1.32), forced expiratory volume in 1 s (FEV1) −1.75 (±1.33) and FEV_1_/forced vital capacity −0.67 (±1.16).

**Discussion:**

The high prevalence of respiratory abnormalities both in symptomatic and asymptomatic patients emphasises that adults with OA need routine long-term pulmonary follow-up. Standardised assessments—including PFTs and CT imaging—may be valuable in lifelong OA care to enable early detection and management of respiratory complications.

**WHAT IS ALREADY KNOWN ON THIS TOPIC**

- Previous studies have described respiratory symptoms and pulmonary function in adults born with oesophageal atresia (OA), but no study has systematically assessed pulmonary outcomes in adulthood, including standardised chest CT, in a large cohort within a structured adult follow-up programme.

**WHAT THIS STUDY ADDS**

- We show a high prevalence of respiratory symptoms and structural abnormalities, including bronchiectasis in asymptomatic patients. Importantly, patient-reported symptoms correlated poorly with pulmonary function and CT findings, highlighting the limitations of symptom-based follow-up and the need for systematic testing.

**HOW THIS STUDY MIGHT AFFECT RESEARCH, PRACTICE OR POLICY**

- Our findings support the implementation of standardised pulmonary follow-up in all adults after OA repair, including pulmonary function test and chest CT at baseline assessment, irrespective of symptoms. Dedicated pulmonary follow-up programmes for adults with OA should be established, ideally including pulmonologists familiar with the long-term sequelae of OA.

## Introduction

 Oesophageal atresia (OA) is a congenital disorder typically characterised by discontinuity of the oesophagus, and accompanied by a trachea-oesophageal fistula (TOF) in 70–90% of cases (see figure 1).^1^ Surgical repair is usually performed shortly after birth, and advances in neonatal surgery and paediatric care have significantly improved survival rates, now exceeding 90%.^1 2^ As a result, an increasing number of individuals with OA are transitioning into adulthood, highlighting a growing challenge in adult care. This shift in clinical focus toward long-term health outcomes represents one of the major challenges for the coming decade in the management of congenital malformations, such as OA.

**Figure 1:**
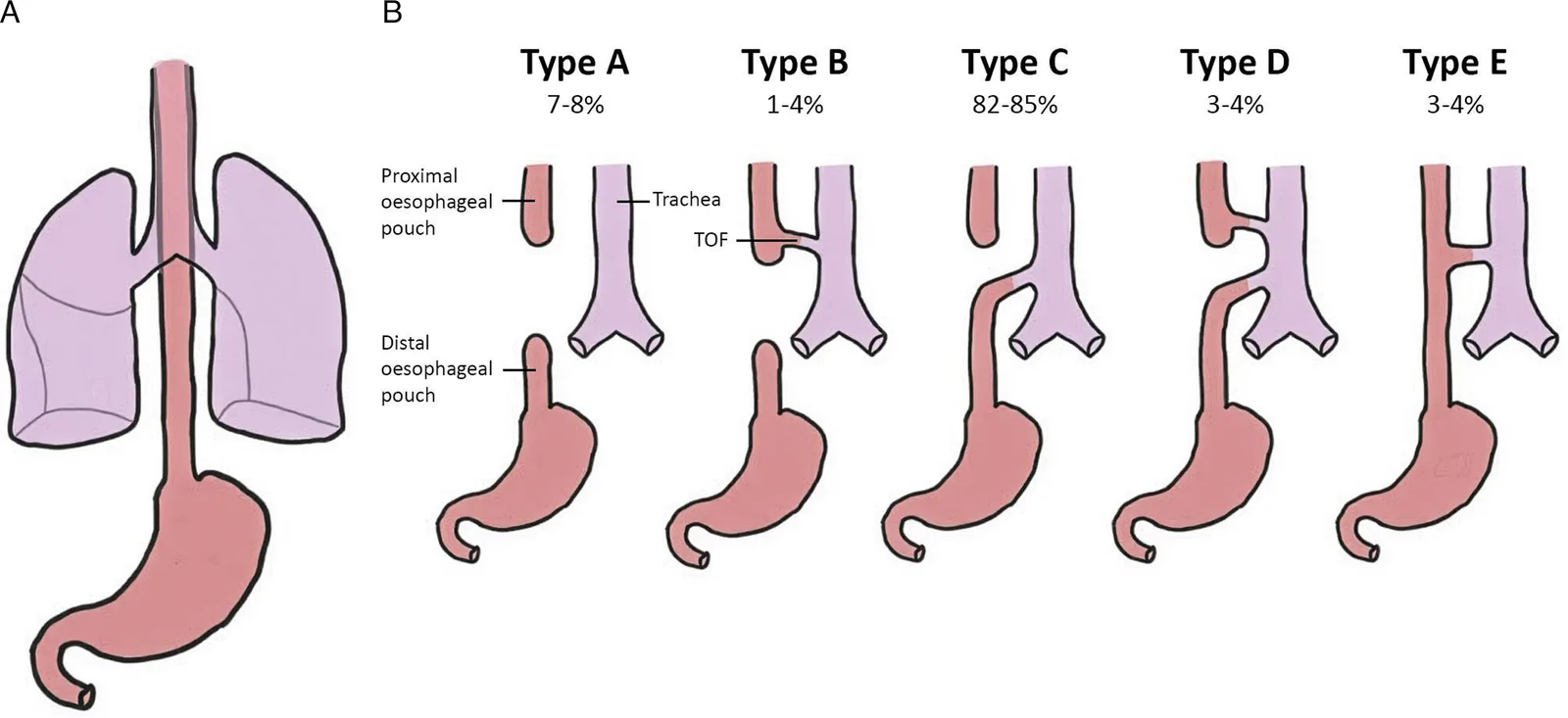
OA types according to Gross classification. (**A**) Normal oesophageal anatomy in which there is an anatomical distinction between the oesophagus and trachea. (**B**) The classification of oesophageal atresia according to Gross: Gross type A; OA without TOF, Gross type B; OA with proximal TOF, Gross type C; OA with distal TOF, Gross type D; OA with distal and proximal TOF and Gross type E; TOF without OA. Percentages indicate the frequency of each subtype. OA, oesophageal atresia; TOF, trachea-oesophageal fistula.

Adult pulmonologists will increasingly encounter and treat these patients, as respiratory comorbidities, such as chronic cough and respiratory tract infections, are frequently reported complications in this population.^2 3^ However, despite the high prevalence of respiratory issues, data on pulmonary outcomes in adults with OA remain scarce, resulting in under-recognition of this group in respiratory medicine.^4^

Although guidelines and expert consensus statements recommend structured pulmonary follow-up—including pulmonary function testing (PFT) and chest CT—implementation in adult OA care remains inconsistent.^3–5^ To our knowledge, no prior studies evaluated the outcomes of structured chest CT imaging in a large cohort of adults born with OA.

This retrospective study presents data from the first consultation of the structured adult OA follow-up programme at Erasmus Medical Center, which since 2019 has included pulmonology consultations, PFTs and chest CT imaging. We report the prevalence of respiratory symptoms, PFTs and radiological abnormalities, and explore associations between these parameters to assess the need for standardised pulmonary surveillance in adults with OA.

## Methods

### Study design and participants

In 2019, the pulmonology department at Erasmus Medical Center, Rotterdam, the Netherlands, implemented a standardised programme to evaluate pulmonary outcomes in adults born with OA. This cross-sectional study using existing data from an OA standardised evaluation programme includes data from patients attending their first consultation between January 2019 and October 2022.

Patients born between 1958 and 2005 with a diagnosis of OA, attending the outpatient clinic and able to undergo routine assessments, were included in this study. Most patients were referred through two structured long-term follow-up programmes: the paediatric surgery long-term follow-up programme for congenital anomalies (initiated in 1999, continuing until age 18) and the adult gastroenterology follow-up programme established in 2013.^6^ As part of the adult programme, all patients with OA, who were born before paediatric follow-up was established, were actively invited based on institutional records. Patients referred from the paediatric follow-up had their first visit at the age of 17, allowing a brief overlap with the adult programme. In 2019, the structured gastroenterology follow-up was extended to the adult pulmonology department, forming the basis of this study. In addition, patients who had surgery at other Dutch paediatric surgery centres were also referred to our expertise centre through the national collaborative network and patient support group for OA. All patients had undergone open surgical repair via right sided thoracotomy. Clinical data—including OA subtype, body mass index, ethnicity, smoking history and inhaled medication use—were extracted from medical records.

### Clinical and diagnostic pulmonary outcomes

All patients were evaluated by a pulmonologist. Respiratory symptoms—including cough, dyspnoea, wheezing, sputum production and exercise-induced problems—were assessed during consultation. No standardised questionnaire beside the regular clinical respiratory symptoms was used; undocumented symptoms were considered absent. Symptom burden was categorised as: none, 1–3 symptoms or ≥4 symptoms.

All patients were offered a non-contrast chest CT scan, consisting of both inspiratory (slice thickness of 1.0 mm) and expiratory acquisitions. Scans were initially reviewed by experienced thoracic radiologists based on a standardised CT-request for OA surveillance. The scans were subsequently re-evaluated by a trained medical student (JFAvdV) using a standardised scoring system, with access to the original radiological report. When uncertainty arose regarding the scoring of a finding, the scan was reviewed and discussed with a thoracic radiologist. The radiologists were blinded to symptoms and PFT, as no additional clinical information was available beyond the standard referral question. However, the medical student had access to the patient files. The structural abnormalities assessed included bronchiectasis, air trapping, lung hypoplasia, tracheomalacia, tracheal diverticula and osseous deformities (eg, scoliosis, rib or vertebral anomalies).

Bronchiectasis was classified as present or absent based on one or more of the following criteria: bronchial dilatation of the bronchus relative to the accompanying pulmonary artery (signet ring sign), lack of bronchial tapering or the presence of visible bronchi within 1 cm of the pleural surface (‘tram-tracking’).^7^ The clinical severity of bronchiectasis was categorised according to the Bronchiectasis Severity Index (BSI).^8^ Air trapping and lung hypoplasia were graded as absent, lobar or generalised. Air trapping was defined as a less-than-normal increase in attenuation on expiratory scan.^7^ Lung hypoplasia was identified by markedly reduced volume of one or both lungs, accompanied by associated findings, such as mediastinal shift, reduced bronchovascular tree, compensatory hyperinflation, a hypoplastic pulmonary artery and reduced perfusion. Tracheomalacia was classified as mild-to-moderate (70–90% reduction in tracheal lumen) or severe (>90% reduction or complete contact between the anterior and posterior tracheal walls), based on manual expiratory scan measurements.^9^

PFTs were performed according to European Respiratory Society (ERS) and American Thoracic Society (ATS) standards.^10^ Spirometry and whole-body plethysmography were conducted preinhalation and postinhalation of 400 µg salbutamol. Forced expiratory volume in 1 s (FEV1), forced vital capacity (FVC) and FEV_1_/FVC were measured via spirometry, while diffusing capacity of the lungs for carbon monoxide (DLCO) was assessed using single-breath techniques. Whole-body plethysmography provided total lung capacity (TLC), residual volume (RV), RV/TLC ratio and specific airway resistance (sRaw). Spirometry parameters were interpreted using the Global Lung Function Initiative (GLI)-2012 reference equations, DLCO using the GLI-2017 reference equations and lung volumes using the European Community for Coal and Steel reference equations.^11–13^ Z-scores were adjusted for sex, age, height and ethnicity. Abnormal PFT results were defined as z-score <−1.64, except for sRaw (>1.64).^10^ Bronchodilator reversibility followed ATS/ERS 2022 criteria: >10% increase in predicted FEV_1_ and/or FVC.^10^

### Data analysis

Depending on data distribution, results were expressed as mean (SD), median (IQR) or counts (%). To evaluate associations between structural CT scan abnormalities and symptom burden or PFT, a proportion score was calculated to correct for missing data: one point per relevant abnormality divided by available variables. Relevant CT abnormalities included bronchiectasis, air trapping, hypoplasia, tracheomalacia, consolidations and nodules.

Associations between continuous and categorical variables were evaluated using independent samples t-tests or analysis of variance (ANOVA), for normally distributed variables and Mann-Whitney U or Kruskal-Wallis tests, for non-normally distributed variables. Post hoc pairwise analyses following significant ANOVA or Kruskal-Wallis tests were adjusted for multiple testing using the Bonferroni correction. χ² tests, with p values estimated by Monte Carlo simulation when expected cell counts were low, were used for two categorical variables. Multivariable logistic regression analyses were performed to evaluate associations between bronchiectasis and relevant clinical variables. To minimise the risk of model overfitting, the number of variables included in the model was restricted according to the recommended minimum of 10 events per variable. Data were analysed using Statistical Package for the Social Sciences (SPSS V.28, IBM, Chicago, Illinois, USA). All tests used a two-sided significance level of 0.05.

### Patient and public involvement

Patients or public were not involved in the design, or conduct, or reporting, or dissemination plans of the research.

## Results

All 182 included patients were seen by a pulmonologist (AJFD, LSK or LSV). One patient did not undergo chest CT imaging. Online supplemental figure 1 shows the inclusion flowchart including how patients were referred to the pulmonary follow-up programme.

### Baseline characteristics

Baseline characteristics are shown in table 1. The mean age at consultation was 33±11 years. Most patients had OA type C (85%) and 17 (9%) had type A.

**Table 1:** Clinical characteristics of the OA population

General characteristics	Patients with OA (n=182)
Female	93 (51)
Age at consultation (years)*	33 (±11)
OA type	
Type A	17 (9)
Type B	1 (1)
Type C	154 (85)
Type D	0
Type E	1 (1)
Unknown	9 (5)
BMI (kg/m²)†	
All	23.4 (20.6–26.0)
Female	23.3 (20.9–25.6)
Male	23.5 (20.4–26.1)
Ethnicity	
Caucasian	171 (94)
African American	2 (1)
North East Asian	0
South East Asian	2 (1)
Mixed	6 (3)
Unknown	1 (1)
Smoking	
Never	133 (73)
In the past	28 (15)
Currently	20 (11)
Unknown	1 (1)
Pack years (years)‡	6.0 (3.0–11.0)
Inhalation medication prescribed	34 (19)

Data are presented as count (percentage) or as otherwise indicated.

* Mean±SD.

† Median (IQR).

‡ Pack years of people who currently smoke and people who previously smoked, median (IQR).

BMI, body mass index; OA, oesophageal atresia.

### Respiratory symptoms

All 182 patients underwent symptom assessment by a pulmonologist. Overall, 46% reported at least one respiratory symptom, with exertional dyspnoea being the most frequently reported symptom (31%) (online supplemental table 1). Cough (27%) and sputum production (11%) were also among one of the more frequently reported symptoms.

### Findings on chest CT

Relevant structural chest CT abnormalities were observed in 128 (71%) patients. Bronchiectasis was present in 12%, and tracheomalacia in 39% of patients, of which 44% were classified as severe. Among patients with bronchiectasis, 17 (77%) patients had mild bronchiectasis, 3 (14%) moderate and 2 (9%) severe bronchiectasis according to the BSI score. The distribution of BSI scores according to age group and respiratory symptom burden is provided in online supplemental table 2. Furthermore, the majority of patients with any kind of TOF (79%) had a tracheal diverticulum. Rib deformities were the most frequently observed osseous abnormality (n=49, 27%), most commonly a rib fusion on the right. Details are summarised in table 2, and representative CT abnormalities are illustrated in figure 2.

**Table 2:** Findings on chest CT of patients with OA

	Patients with OA (n=181)
	Count (%)
**Pulmonary abnormalities**	
Bronchiectasis:	
None	159 (88)
Present	22 (12)
Air trapping*:	
None	108 (62)
Lobar	51 (29)
Generalised	16 (9)
Lung hypoplasia areas:	
None	154 (85)
Local	25 (14)
Generalised	2 (1)
**Tracheal abnormalities**	
Tracheomalacia*:	
None	109 (62)
Mild/moderate	38 (22)
Severe	29 (17)
Tracheal diverticulum†	130 (79)
**Osseous abnormalities** ‡	
Scoliosis	28 (16)
Rib deformities	49 (27)
Vertebral deformities	14 (8)

Data are shown as count (percentage). We labelled a chest CT to contain lung hypoplasia: if lung tissue was missing, but it was not caused by iatrogenic actions, such as a lobectomy.

* In five patients no (adequate) expiration images were made, hence it was impossible to evaluate if tracheomalacia or air trapping was present. In one additional patient measurements of air trapping was not possible, because of suboptimal expiratory effort.

† N=164, corrected for 17 patients without any tracheo-oesophageal fistula.

‡ Multiple options per patient possible.

OA, oesophageal atresia.

**Figure 2:**
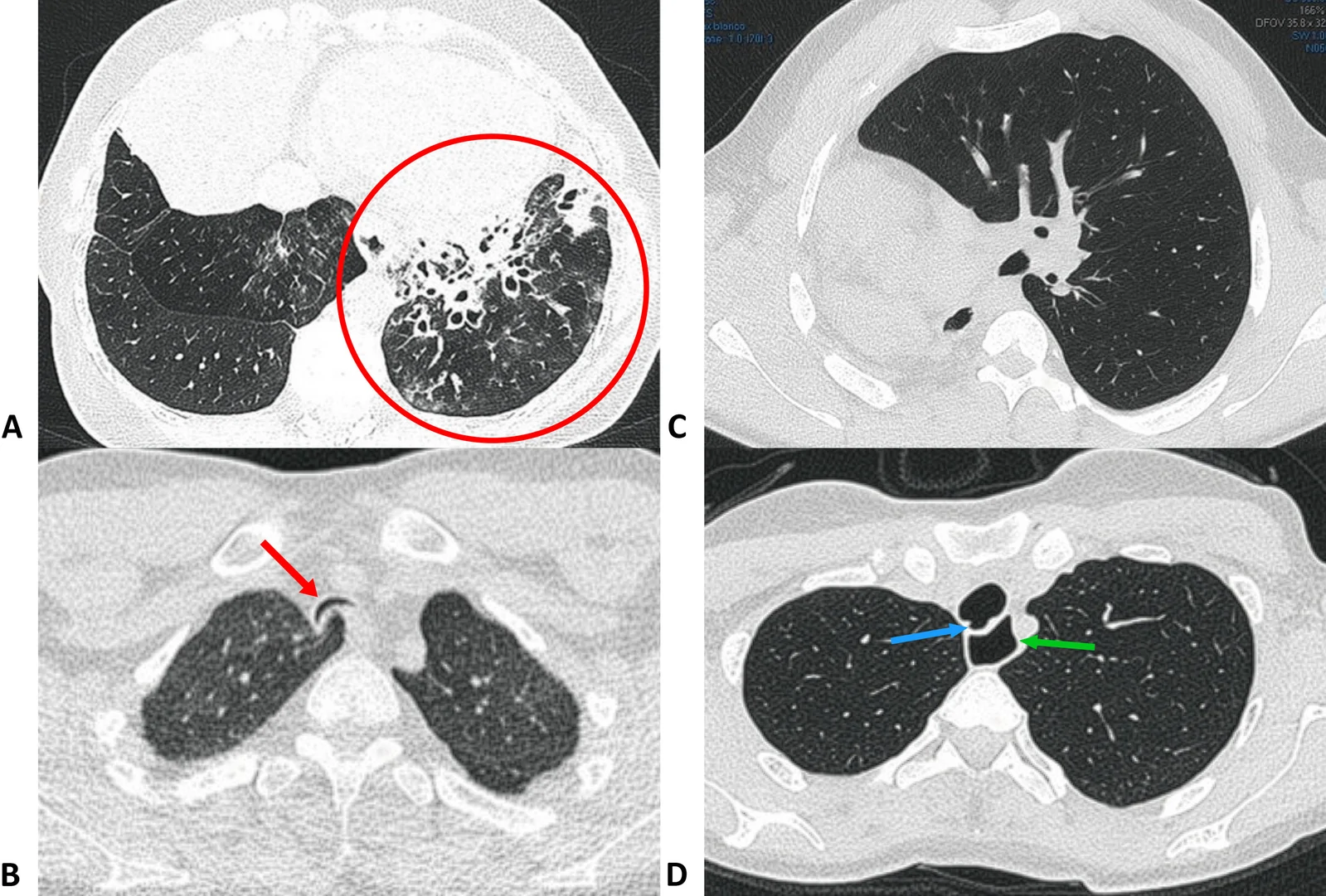
Abnormalities seen on chest CT imaging. (**A**) Bronchiectasis (within red circle) with associated bronchial wall thickening and peribronchial consolidation in the left lower lobe. (**B**) Severe tracheomalacia (red arrow) demonstrating the characteristic ‘frown face’ appearance on expiratory imaging. (**C**) Right lung agenesis with marked mediastinal shift and compensatory hyperinflation of the left lung. (**D**) Small posterior residual tracheal diverticulum (blue arrow) following TOF repair, accompanied by oesophageal dilatation (green arrow). TOF, tracheo-oesophageal fistula.

### Pulmonary function testing

Table 3 summarises the PFT results. All mean z-scores for PFT parameters were statistically below reference mean (z-score=0). Of all patients with OA, 126 (69%) had at least one abnormal PFT parameter (z-score <−1.64), including reduced FEV_1_ in 86 (47%) patients and reduced FEV_1_/FVC in 38 (21%). Among these 38 patients, postbronchodilator PFTs were available for 28 patients, of whom 19 (68%) had irreversible airflow obstruction. A decreased TLC was also frequently observed (n=69, 39%).

**Table 3:** Results of pulmonary function test of patients with OA

		Patients with OA (n=182)	
		Mean (SD)	Patients with abnormal z-score (%)*
Prebronchodilator			
TLC†	(n=177)	−1.34±1.32	69 (39)
FVC	(n=182)	−1.46±1.33	73 (40)
FEV_1_	(n=182)	−1.75±1.33	86 (47)
FEV_1_/FVC	(n=182)	−0.67±1.16	38 (21)
RV	(n=177)	−0.58±0.98	21 (12)
RV/TLC	(n=177)	−0.02±1.33	5 (3)
sRaw	(n=177)	1.41±1.79	61 (35)
Postbronchodilator			
TLC†	(n=169)	−1.32±1.33	67 (40)
FVC	(n=170)	−1.39±1.32	60 (35)
FEV_1_	(n=140)	−1.44±1.40	55 (39)
FEV_1_/FVC	(n=140)	−0.05±1.13	11 (8)
RV	(n=169)	−0.66±0.94	24 (14)
RV/TLC	(n=169)	−0.14±1.23	6 (4)
sRaw	(n=167)	0.30±1.27	20 (12)
Diffusion capacity			
DLCO_C_	(n=166)	−0.77±1.15	31 (19)

Data are presented as number of patients, mean±SD of z-score and count (percentage). In all 182 patients, pulmonary function testing was performed. However, not all pulmonary function parameters were available in these patients. The FVC, FEV_1_ and FEV_1_/FVC prebronchodilator were available in 182 patients while the FEV_1_, and FEV_1_/FVC postbronchodilator were available in 140 patients.

* Abnormal z-score is defined as <−1.64, except for sRaw (>1.64).

† TLC was measured in whole-body plethysmography.

DLCO_c_, diffusing capacity of the lungs for carbon monoxide corrected for hemoglobin level; FEV_1_, forced expiratory volume in 1 s; FVC, forced vital capacity; OA, oesophageal atresia; RV, residual volume; sRaw, specific airway resistance; TLC, total lung capacity.

### Association between symptoms, chest CT abnormalities and pulmonary function testing

Patients reporting more symptoms had a significantly higher proportion of CT abnormalities (p=0.022). Post hoc analysis showed asymptomatic patients had a significantly lower mean proportion of CT abnormalities (mean±SD: 0.17±0.17), compared with those reporting one to three symptoms (0.24±0.18; p=0.032, Bonferroni-corrected). However, 62% of asymptomatic patients still showed CT abnormalities. A normal FEV_1_ was associated with a significantly higher prevalence of a normal CT-scan (p<0.001). Nevertheless, 64% of patients with a normal FEV_1_ still had at least one relevant CT-abnormality. This association was not observed in patients with an obstructive lung function (p=0.889), as both 71% of patients with a normal FEV_1_/FVC z-score and 71% of patients with an obstructive lung function had at least one relevant CT-abnormality.

Bronchiectasis occurred across all age groups (online supplemental figure 2). It was present in 11 of 52 patients (21%) reporting cough and/or sputum production versus 11 of 129 patients (9%) of those without symptoms (p=0.019, online supplemental figure 3A). After adjustment for smoking habit status, cough and/or sputum production remained significantly associated with bronchiectasis (OR 1.94, 95% CI 1.08 to 3.48, p=0.027). Bronchiectasis severity according to the BSI score was also significantly higher in patients with these symptoms compared with asymptomatic patients (p=0.036). Prebronchodilator and postbronchodilator FEV_1_ values were significantly lower in patients with bronchiectasis than in patients without bronchiectasis (prebronchodilator mean±SD: −2.79±1.40 vs −1.62±1.27, p<0.001; postbronchodilator mean±SD: −2.83±1.29 vs −1.28±1.32, p<0.001). The prevalence of bronchiectasis did not differ significantly between patients with and without tracheomalacia (respectively 6 patients (9%) vs 16 (15%), p=0.265).

Furthermore, no significant associations were observed between tracheomalacia and symptom burden (p=0.425, online supplemental figure 3B), nor between tracheal diverticula and the number of symptoms (p=0.780, online supplemental figure 3C).

An additional analysis examined the relationship between respiratory symptom burden and PFT results. Reduced FEV_1_ was observed in 86 patients (47%), who had significantly more symptoms: 48 (56%) reported one to three symptoms and 4 (5%) reported ≥4 symptoms, compared with 31 (32%) and none, respectively, among patients with normal FEV_1_ (p<0.001). Among the 38 patients with obstructive lung function, 18 (48%) reported one to three symptoms, versus 61 (42%) of those with a normal FEV_1_/FVC-ratio (p=0.264). No significant association was found between airflow obstruction and tracheomalacia (p=0.427).

## Discussion

This retrospective study demonstrates a high prevalence of respiratory morbidity in adults with OA, indicating the potential value of structured pulmonary follow-up in adulthood in this population. Nearly half of the patients (46%) reported respiratory symptoms, while abnormalities were identified in 69% of PFTs and 71% of chest CT scans. Importantly, structural abnormalities on CT were present in 62% of asymptomatic patients, underscoring the limited predictive value of symptoms alone in identifying underlying pathology. Bronchiectasis was observed in 12% of patients, occurring across all age groups, including asymptomatic patients. This finding suggests that symptom burden may not reliably reflect the underlying structural abnormalities, supporting the inclusion of chest CT imaging in baseline respiratory assessment.

Exertional dyspnoea (31%), cough (27%) and sputum production (11%) were the most frequent respiratory symptoms. This prevalence aligns with Chetcuti and Phelan, who reported persistent symptoms in 57% of patients with OA aged ≥15 years, but exceeds the 32–33% reported by Koivusalo *et al* and Brooks *et al.*^14–16^ Symptom underreporting likely contributes to these discrepancies, as many patients habituate to chronic symptoms from early childhood.^15^ Notably, the high prevalence of exertional dyspnoea in our cohort is consistent with findings from Koivusalo *et al*, who reported that 33% of patients with OA experience impaired physical performance.^15^ Although most patients (85%) report good quality of life, and only 5% attribute this reduction specifically to respiratory symptoms. These findings suggest that subjective symptom burden underestimates the true extent of pulmonary pathology in this population.

Recurrent lower respiratory infections are common, particularly in early life. Approximately 31% of patients with OA develop pneumonia in the first 5 years, declining to 5% after age 15.^14^ Asemota *et al* reported similarly elevated rates of lower respiratory infections and concluded that respiratory morbidity decreases with age.^17^ However, their analysis relied on health records and lacked patient-reported outcomes, PFTs or imaging data. Conversely, our findings suggest that structural abnormalities can persist or even progress subclinically. Notably, 62% of asymptomatic patients in our cohort exhibited structural abnormalities on chest CT, underscoring the importance of incorporating routine imaging into long-term follow-up protocols, regardless of symptoms.

The most frequently observed CT abnormalities were tracheal diverticulum (79%), tracheomalacia (39%) and air trapping (38%). Additional findings included lung hypoplasia (15%) and bronchiectasis (12%), highlighting the complex pulmonary phenotype in adults with corrected OA.

Bronchiectasis was present in 12% of patients, a markedly higher prevalence than in the general population (50–1000 cases per 100 000).^18^ This aligns with the 17% reported by Banjar, in a mixed-age cohort.^19^ The higher prevalence of 31% reported by Porcaro *et al* likely reflects their focus on symptomatic children.^20^ Our unstratified adult cohort may have included more asymptomatic or mildly affected individuals, accounting for the lower prevalence. Moreover, most bronchiectasis cases were classified as mild according to the BSI score, although 23% of affected patients had moderate or severe disease. Importantly, bronchiectasis was significantly more frequent in symptomatic patients (21%) compared with asymptomatic patients (9%), and was observed across all age groups. This association remained significant after adjustment for smoking status. Tracheomalacia and bronchiectasis may have overlapping pathophysiological mechanisms, as tracheomalacia can lead to sputum stasis resulting in recurrent infections.^21^ However, in our cohort, no significant association was observed between bronchiectasis and tracheomalacia. These observations underscore the potential value of pulmonary follow-up in adulthood, as bronchiectasis is known to be progressive and associated with recurrent infections and long-term respiratory decline, highlighting the importance of early detection, particularly because structural abnormalities may be present even in asymptomatic patients.^22^

In patients with OA, bronchiectasis may result from chronic aspiration or recurrent lower airway infections. Gastro-oesophageal reflux disease, which is common in this population, may contribute through microaspiration—often unnoticed by patients.^23^ These findings support inclusion of chest CT in routine follow-up, even in asymptomatic patients. Based on our results, and given that bronchiectasis occurred in 14% of patients aged 17–20 years old, our paediatric OA follow-up protocol has been modified to include chest CT at age 8, enabling earlier detection and intervention. Evaluation of this early chest CT is needed to optimise care during adolescence and improve our understanding of the pathogenesis and timing of bronchiectasis development.

Tracheal diverticulum was the most common CT finding, observed in 79% of patients. These diverticula represent remnants of the TOF following surgical ligation, during which the surgeon aims to make this remnant as short as possible.^24^ Although typically asymptomatic, they may contribute to secretion retention, infection and chronic cough.^25^ However, we found no significant association between tracheal diverticula and cough or sputum production. These findings do not provide evidence to support routine surgical intervention. Nevertheless, tracheal diverticula should be considered a possible contributor in the differential diagnosis of persistent or unexplained respiratory symptoms.

Tracheomalacia occurred in 39% of patients, lower than the 64–70% prevalence reported in adolescents by Peetsold *et al*, but higher than the 10–13% estimated in adolescents and adults.^3 26^ This discrepancy likely reflects both age-related changes and differences in diagnostic methodology, as the clinical relevance of tracheomalacia tends to decline with age.^3^ Peetsold *et al* defined tracheomalacia clinically by barking cough, whereas we used expiratory CT scans and stricter radiological criteria (≥70% airway collapse), consistent with evidence showing that up to 50% collapse can be observed in healthy individuals.^9 26^ To our knowledge, no previous adult OA studies have reported tracheomalacia prevalence using imaging. Although bronchoscopy remains the diagnostic gold standard, despite its accuracy depending on operator experience, prior studies have shown good correlation with expiratory CT imaging.^27^

Other CT abnormalities included scoliosis (16%), rib deformities (27%) and vertebral anomalies (8%), consistent with previous reports, including Mishra *et al*, who reported scoliosis in 20% of both paediatric and adult patients.^28^ Secondary scoliosis may result from right thoracotomy, the standard surgical approach for OA repair in our patients, while primary scoliosis may reflect underlying congenital vertebral anomalies, for example, as seen in VACTERL association.^28 29^ Thoracoscopic surgical approaches, although less extensively studied, may reduce long-term musculoskeletal sequelae such as rib deformities and restrictive lung changes.^28^

Pulmonary function was significantly impaired in our cohort, consistent with previous research. Sistonen *et al* found at least one abnormal PFT parameter in 78% of patients, closely aligning with our finding of 69%.^30^ Given the high prevalence of abnormalities and accessibility of PFT, routine inclusion of PFTs in adult OA follow-up is strongly recommended.

An airflow obstruction occurred in 21–30% of patients in earlier studies, comparable to our finding.^30–32^ However, bronchodilator reversibility was noted in only 21% of cases by Gatzinsky *et al*, compared with 32% in our cohort.^31^ This discrepancy may partly reflect a stricter z-score of <−1.96 for obstruction compared with our z-score of <−1.64, in accordance with the ERS/ATS guideline.^10^ In our study, postbronchodilator data were not available for all patients due to COVID-19-related logistic restrictions and infection control measures, which limited testing capacity and reduced feasibility of full PFT. This may have influenced results.

A reduced FEV_1_ was seen in 47% of our cohort, in line with prior studies showing a decreased FEV_1_ in 50% of adult patients with OA.^31^ Notably, 30% of 8 years old patients with OA had an abnormal FEV_1_, suggesting deterioration over time.^33^ Interestingly, in contrast to prior cohorts, we observed significant associations of both bronchiectasis and respiratory symptoms with lower FEV₁ values (prebronchodilation and postbronchodilation), likely reflecting increased statistical power in our larger sample.^30–32^ These associations underscore the potential value of PFTs not only for functional assessment, but also for revealing structural pulmonary abnormalities in asymptomatic patients. Similarly, no significant difference in CT abnormalities was observed between patient with normal versus obstructive FEV_1_/FVC-values, highlighting that normal lung function does not exclude structural pulmonary disease.

To our knowledge, this is the largest study to date assessing pulmonary CT abnormalities in adult patients with OA at enrolment in a follow-up programme. Previous studies reported imaging findings, but were limited by small sample sizes and narrower diagnostic protocols.^19 20^ Conversely, our study integrates CT imaging, PFTs and symptom data, providing a comprehensive assessment of structural abnormalities, pulmonary function impairment and clinical concerns.

Our findings emphasise that respiratory morbidity persists into adulthood, even in asymptomatic individuals and that symptoms associate poorly with structural findings. This highlights the need for structured, multidisciplinary surveillance. We advocate for the establishment of dedicated adult OA care teams including pulmonologists familiar with OA-related long-term sequelae, to ensure timely recognition and management of respiratory complications in patients with OA. In addition, it is essential to raise awareness among adult pulmonologists, who will encounter patients with OA more frequently given the growing number of adults with OA. They should be made aware of the long-term respiratory risks associated with OA, to ensure accurate diagnosis and management of persistent respiratory symptoms.^34^

The main limitation of our study is its cross-sectional design, capturing data only at the initial visit within a longitudinal follow-up programme. Consequently, it does not provide insight into disease progression or treatment outcomes over time. Future research should assess whether structured surveillance reduces respiratory morbidity and improves respiratory outcomes in adulthood and establish evidence-based strategies regarding the timing and frequency of follow-up assessments, including chest CT imaging and PFT. Additionally, future studies should consider gastrointestinal symptoms, as these may also contribute to respiratory morbidity. Nonetheless, in the context of limited respiratory data in adults with OA, our findings offer valuable insights into the burden of structural and functional pulmonary abnormalities in this population. Following the initial consultation, each patient enters a personalised follow-up programme based on the initial findings, with a maximum interval of 5 years.

15 patients (7%) were excluded due to missed or cancelled consultations, potentially introducing limited selection bias. In addition, patients entered the follow-up programme through different referral pathways. While referral through our structured follow-up programme was standardised, other referral routes may have resulted in some degree of selection bias. Although the proportion of non-participants was small, the study population may not be fully representative of all adults with OA. For instance, non-attending patients might have been largely asymptomatic, potentially leading to a slight overestimation of abnormalities. On the other hand, symptom assessment was not standardised, and undocumented symptoms were considered absent, potentially resulting in an underestimation of symptom burden and misclassification of patients as asymptomatic. Future prospective studies in children and in adults should include a standardised recording of symptoms. Nevertheless, a high prevalence of abnormalities on chest CT and PFT was observed within the cohort. Moreover, as previously mentioned, patients may become habituated to their symptoms, under-reporting them despite structural or functional abnormalities on chest CT or PFT. These considerations emphasise the importance of informing patients and their caregivers about the purpose and significance of follow-up in adulthood.

## Conclusion

Our study underscores the high prevalence of respiratory symptoms and structural lung abnormalities in adults following surgical repair of OA. Given the poor association between symptoms and imaging findings, we support the inclusion of standardised pulmonary follow-up—including PFT and chest CT imaging during baseline assessment—in care pathways for adults born with OA. As more individuals with OA reach adulthood, adult pulmonologists will increasingly encounter these patients and be responsible for managing their respiratory health. Multidisciplinary follow-up programmes, involving specialists familiar with OA-related long-term effects, are essential for earlier intervention, improved symptom control and ultimately better respiratory outcomes in adulthood. These findings from the first pulmonary visit highlight the potential value of our dedicated adult follow-up programme and provide a foundation for establishing an optimised surveillance programme for adults with OA. Future prospective studies are needed to evaluate the impact of structured pulmonary follow-up on patient outcomes and to determine optimal follow-up strategies, including the frequency and timing of repeated PFT and chest CT imaging.

## Supplementary material

10.1136/bmjresp-2026-004434 online supplemental file 1

## Data Availability

Data are available upon reasonable request.
