# Supplementary material for: High prevalence of pulmonary abnormalities in adults with oesophageal atresia: findings from a structured follow-up programme
Source: BMJ Open Respir Res. 2026 Nov 9;13(1):e004434. doi: 10.1136/bmjresp-2026-004434 (PMC13756223; doi:10.1136/bmjresp-2026-004434)
Supplement: Supplementary file 1 [file bmjresp-13-1-s001.pdf]

SUPPLEMENTAL MATERIAL

Wintels et al. - High Prevalence of Pulmonary Abnormalities in Adults with Oesophageal Atresia: Findings from a Structured Follow-up Programme

Supplementary Figure 1. Patient inclusion and referral flowchart

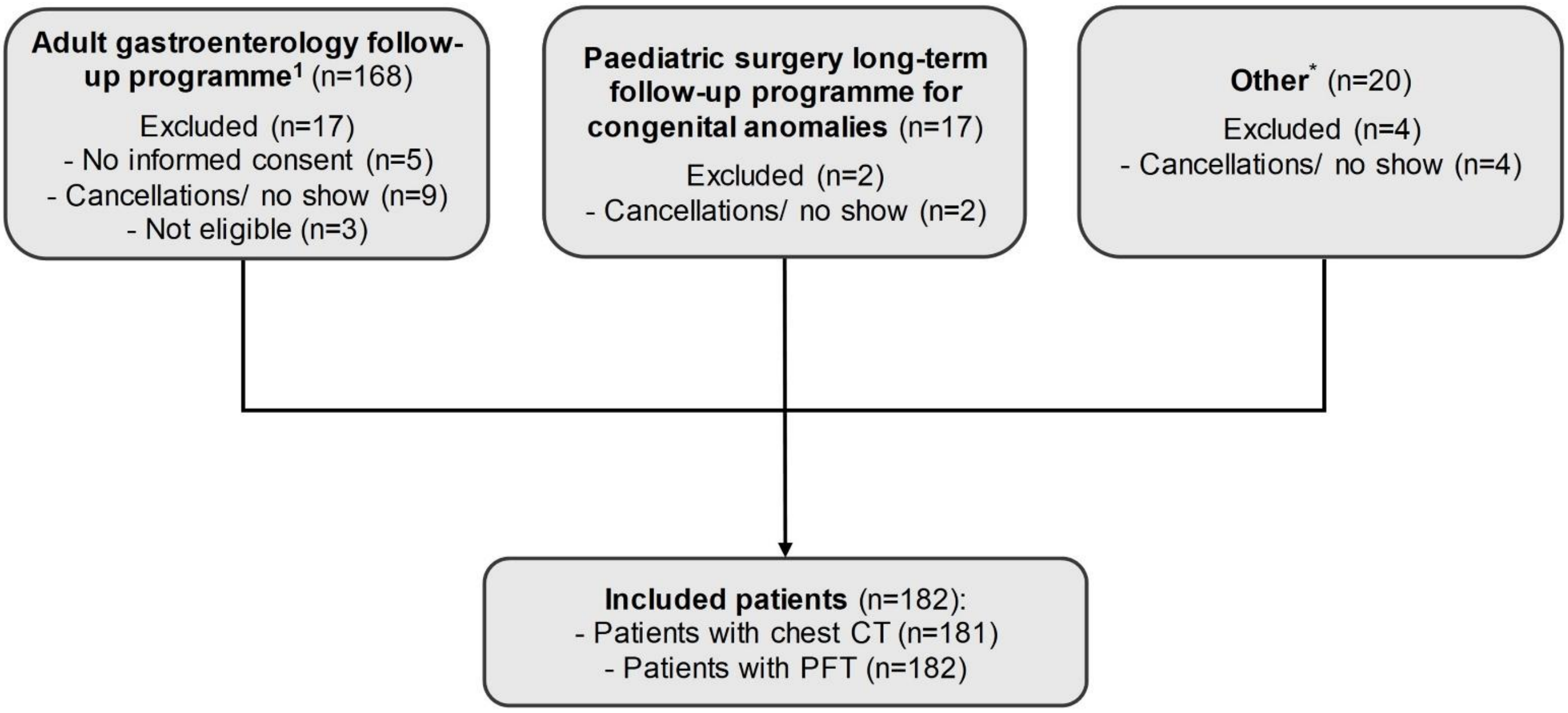

Most patients (N=168) were referred from the adult gastroenterology follow-up programme established in 2013. As part of the adult programme, all OA patients, who were born before paediatric follow-up was established, were actively invited based on institutional records. In 2019, the structured gastroenterology follow-up was extended to the adult pulmonology department. Patients in paediatric surgery long-term follow-up will be referred to the adult pulmonology department at age 17 years. All included patients (n=182) had pulmonary function tests performed, and chest CT was missing for one patient. Three patients were not eligible because both pulmonary function tests and chest CT imaging were of insufficient quality. CT = computed tomography; PFT = pulmonary function testing.

**Other = Referral from an external hospital, referral by a general practitioner, or internal referral within the pulmonology department.*

1. Vergouwe FWT, IJsselstijn H, Biermann K, et al. High Prevalence of Barrett's Esophagus and Esophageal Squamous Cell Carcinoma After Repair of Esophageal Atresia. Clin Gastroenterol Hepatol. 2018;16(4):513-21 e6.

Supplementary Table 1: Respiratory symptoms of OA patients

	**OA patients (n=182)**
**Respiratory symptoms**	**Count (%)**
Dyspnoea:	
At rest	4 (2%)
During exercise	56 (31%)
Wheezing:	
At rest	3 (2%)
During exercise	10 (6%)
Cough	49 (27%)
Sputum production	20 (11%)
Amount of respiratory symptoms per patient (out of 6 symptoms)	
0 symptoms	99 (54%)
1-3 symptoms	79 (43%)
≥4 symptoms	4 (2%)

Data are shown as count (percentage). OA = oesophageal atresia. Respiratory symptoms reported by patients during symptom assessment by the pulmonologist. Undocumented symptoms are reported as absent.

Supplementary Table 2: Bronchiectasis Severity Index (BSI) scores across age groups and respiratory symptoms

Patients with bronchiectasis (n=22)			
	**Mild bronchiectasis**	**Moderate bronchiectasis**	**Severe bronchiectasis**
Age (years):			
17-20	4 (100%)	-	-
21-30	4 (80%)	1 (20%)	-
31-40	2 (67%)	1 (33%)	-
41-50	6 (75%)	1 (13%)	1 (13%)
51-60	1 (50%)	-	1 (50%)
Amount of respiratory symptoms (out of 6 symptoms):			
No symptoms	7 (100%)	-	-
1-3 symptoms	10 (71%)	3 (21%)	1 (7%)
≥ 4 symptoms	-	-	1 (100%)

In total, 22 patients (12%) had bronchiectasis on chest CT. Bronchiectasis severity was categorised according to the Bronchiectasis Severity Index: mild (1-4 points), moderate (5-8 points), or severe (≥9 points).[2] The amount of respiratory symptoms was based on the presence of cough, dyspnoea, wheezing, sputum production, and exercise-induced complaints as reported during symptom assessment by the pulmonologist. Undocumented symptoms were considered absent.

2. Chalmers JD, Goeminne P, Aliberti S, et al. The bronchiectasis severity index. An international derivation and validation study. Am J Respir Crit Care Med. 2014;189(5):576-85.

Supplementary Figure 2. Prevalence of bronchiectasis on chest CT according to age

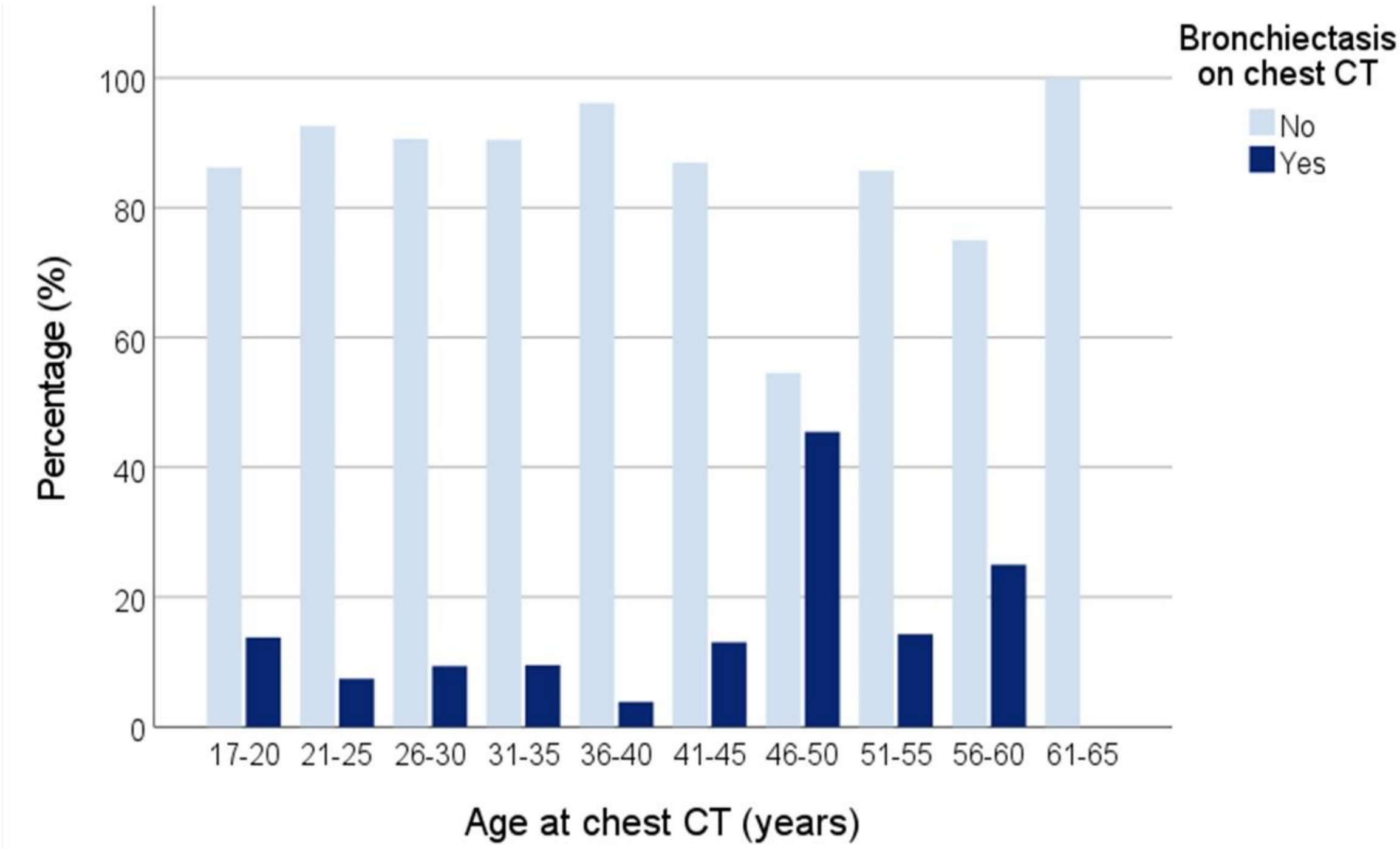

Counts per age subgroup: 17–20 years (n=29), 21–25 years (n=27), 26–30 years (n=32), 31–35 years (n=21), 36–40 years (n=26), 41–45 years (n=23), 46–50 years (n=11), 51–55 years (n=7), 56–60 years (n=4), 61–65 years (n=1).

Supplementary Figure 3. Association between chest CT abnormalities and respiratory symptoms

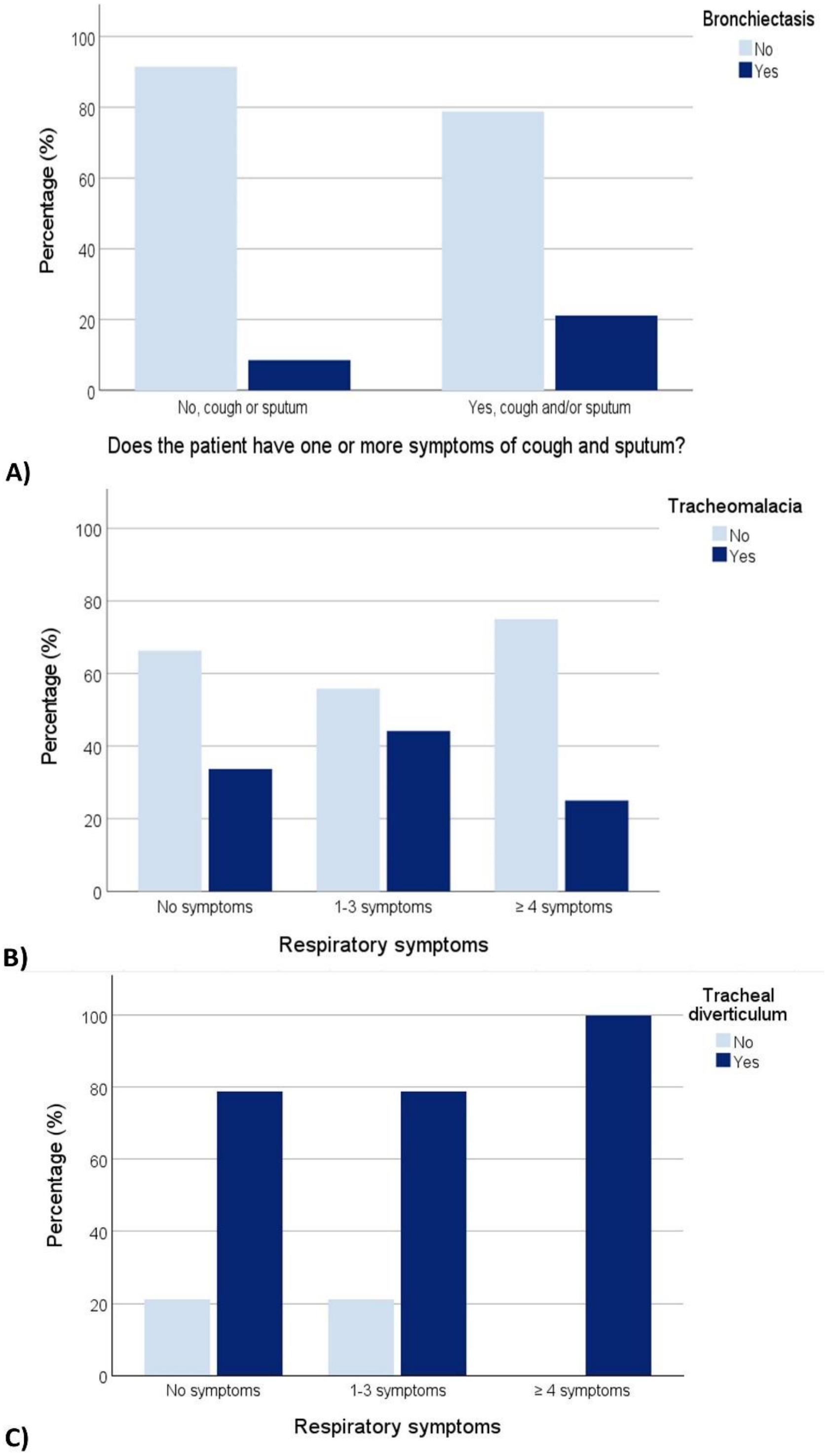

A) The presence of bronchiectasis on chest CT compared to respiratory symptoms of cough and/or sputum.

B) The presence of tracheomalacia on expiratory chest CT compared to respiratory symptoms.

C) The presence of tracheal diverticulum on chest CT compared to respiratory symptoms. Results are presented as percentage within the symptom category and include only patients with a history of tracheo-oesophageal fistula (n=164).
